# Native and tagged CENP-A histones are functionally inequivalent

**DOI:** 10.1186/s13072-024-00543-9

**Published:** 2024-06-02

**Authors:** Minh Bui, Songjoon Baek, Reda S. Bentahar, Daniël P. Melters, Yamini Dalal

**Affiliations:** Center for Cancer Research, Laboratory of Receptor Biology and Gene Expression, National Cancer Institute, 41 Medlars Drive, Bldg 41/Rm B1300, Bethesda, MD 20892 USA

**Keywords:** Epitope tags, Centromere, Chromatin, Post-translational modifications, Chromatin immuno-precipitation, Deep sequencing, Atomic force microscopy, Immuno-fluorescence, CENP-A

## Abstract

**Background:**

Over the past several decades, the use of biochemical and fluorescent tags has elucidated mechanistic and cytological processes that would otherwise be impossible. The challenging nature of certain nuclear proteins includes low abundancy, poor antibody recognition, and transient dynamics. One approach to get around those issues is the addition of a peptide or larger protein tag to the target protein to improve enrichment, purification, and visualization. However, many of these studies were done under the assumption that tagged proteins can fully recapitulate native protein function.

**Results:**

We report that when C-terminally TAP-tagged CENP-A histone variant is introduced, it undergoes altered kinetochore protein binding, differs in post-translational modifications (PTMs), utilizes histone chaperones that differ from that of native CENP-A, and can partially displace native CENP-A in human cells. Additionally, these tagged CENP-A-containing nucleosomes have reduced centromeric incorporation at early G1 phase and poorly associates with linker histone H1.5 compared to native CENP-A nucleosomes.

**Conclusions:**

These data suggest expressing tagged versions of histone variant CENP-A may result in unexpected utilization of non-native pathways, thereby altering the biological function of the histone variant.

**Supplementary Information:**

The online version contains supplementary material available at 10.1186/s13072-024-00543-9.

## Background

The original breakthrough in tagging proteins for visualization inside cells is the ubiquitously used Green Fluorescent Protein (GFP), which was first discovered in 1962 in jellyfish *Aequorea Victoria* [1], crystalized in 1974 [2], cloned in 1992 [3], and successfully expressed and fluoresced in bacteria and worms in 1994 [4]. Later, its stability was improved in 1998 [5]. Fittingly, the Nobel Prize in Chemistry was awarded to Osamu Shimomura, Martin Chalfie, and Roger Tsien in 2008, almost fifty years after its discovery. Since then, numerous types of epitope, short amino acid, and full-length protein tags have been developed and cloned into any gene of interest, later to be expressed and translated for downstream applications such as purification or visualization with highly specific antibodies. Short peptide tags often used for biochemical purifications due to their high immuno-affinity and specificity include FLAG, S-tag, and HA [6, 7]. Since the discovery of GFP, other fluorescent tag derivatives evolved including RFP, YFP, Cerulean, and mCherry, which have been instrumental for cytological FRET studies [8, 9]. Other tags often used for bulk purification of proteins in either mammalian or bacterial expression systems utilize streptavidin or 6XHis, due to the ease of creating resins that can enrich for these tags [6, 10].

To study dynamics of nuclear proteins, researchers have taken advantage of this powerful tool and made great strides in the understanding of chromatin-based nucleosomal dynamics. One example is the study of the centromeric histone H3 variant, CENP-A/CENH3, which structurally and epigenetically marks centromeres in nearly every species studied thus far. Numerous landmark studies have utilized epitope tags for technical purposes, including protein visualization [11–13], live-cell imaging [14, 15], chromatin immuno-precipitation (ChIP) [11, 12], and in vitro protein purification [16], bringing insight into this fascinating protein and its involvement in mitosis.

One of the earliest reports added a nine amino acid C-terminal HA-tag to human CENP-A in immuno-fluorescence (IF) experiments, revealing that the histone fold domain specified centromere-specific deposition, and that over-expression led to promiscuous assembly to non-centromeric regions [17]. Use of a TAP-tagged fly CENP-A (Cid) determined that its chaperone is RbAp48, and that its over-expression also led to promiscuous assembly at non-centromeric regions [18]. In yeast, N-terminally FLAG-tagged CENP-A (Cse4) expressed in a Cse4 knock-out background, revealed that Scm3 is FLAG-CENP-A’s centromere-specific chaperone [19]. Several reports indicated centromeric CENP-A identity was specified by the chaperone HJURP, a mechanism not observed for histone H3 [20–22]. Once at the centromere, FLAG- or GFP-tagged CENP-A (Cid) alternates with histone H3 domains along chromatin fibers [23]. Fluorescently labeled yeast CENP-A (Cse4) also determined CENP-A protein copy number at centromeres, during metaphase and anaphase [13]. Studies examining the impact of CENP-A lysine residue 124 acetylation [12] and ubiquitylation [24] utilize both short epitope and fluorescent tags to determine molecular interactions and localization within the nucleus. All these studies have one common characteristic: they all utilized an epitope tag to elucidate function.

Though popular and technically powerful for scientific insights into otherwise intractable problems in cell biology, tags come with their own drawbacks. For example, though mouse mutants homozygous for CENP-A-GFP exhibited centromere-specific fluorescence, embryos suffered from chromosome mis-segregation, aneuploidy, and apoptosis, ultimately resulting in death [25]. Ravi et al. previously demonstrated that addition of an N-terminal GFP-tag to an H3.3 N-terminal tail + CENP-A (CenH3) chimera generated uniparental haploid plants [26]with severe infertility [27]. In maize, over-expression of CENP-A (CenH3) was lethal, but overexpression of GFP-CenH3 or CenH3-YFP was not and led to centromere expansion without impacting plant growth [28]. Native untagged CENP-A (CenH3) from other plant species were better capable of complementing CENP-A (CenH3) function in *Arabidopsis* than GFP-tagged CENP-A [29, 30]. Though severe meiotic defects appear prevalent in different organisms, little is known about how tagged CENP-A proteins impact post-translational modifications (PTMs), protein-protein interactions, chaperone recruitment, and whether cells prefer native or tagged CENP-A. In this study, we examined these questions using a well-documented C-terminally TAP-tagged CENP-A (CpA-TAP; we will use CpA to refer to experimental samples or conclusions, and CENP-A to refer to the overall gene/protein), transiently expressed at low levels in the presence of native CENP-A (CpA) in human cells. To our surprise, we observed that in competition with native CpA, CpA-TAP binds poorly to CENP-C (its inner kinetochore partner), has altered post-translational modifications, and utilizes the DAXX-mediated and transcription coupled H3.3 chaperone to deposit CpA-TAP at non-centromeric sites. We also examined the impact of an N-terminally tagged GFP-CpA and observed its centromeric disposition is diminished compared to native CpA, but less severe than CpA-TAP. Interestingly, knock-in of the C-terminal TAP-tag into the endogenous CENP-A locus only gave rise to viable heterozygous cells in the DAXX knock-out cell line, but CpA-TAP was eventually silenced while native CpA was solely expressed. These data suggest tagged CENP-A has the potential to exploit similar biological pathways previously reported in CENP-A over-expressing cancer cells [31] by utilizing alternative chaperones, and that tagged CENP-A may not serve as the most effective proxy for native CENP-A dynamics at human centromeres.

## Methods

### Transient transfection and cell lines

CENP-A-TAP (CpA-TAP) driven by CMV promoter plasmid was generously provided by Dan Foltz. Transfections were done using the Neon Transfection System (ThermoFisher Scientific Cat #MPK5000) with 100 uL kit (ThermoFisher Scientific Cat #MPK10096), using the following parameters for both HeLa and HeLa + DAXX Crispr’d KO cell lines: 2 pulses of 1050 V/30 ms. HeLa cells or DAXX CRISPR KO cell line was acquired from AbCam (Cat #ab265233). Cells were harvested 72-hrs post-transfection for down-stream applications.

### Immunofluorescence

For complete procedure, please refer to (11). Mouse anti-S-tag (Millipore Cat #MAC112) and guinea pig anti-CENP-C (MBL Cat #PD030) antibodies were used to detect CpA-TAP and CENP-C, respectively.

### Chromatin immuno-precipitation (chip), chipseq, tau electrophoresis & western/tau-western

For complete native (unfixed) ChIP procedure, please refer to (11) but using DynaBeads (ThermoFisher Cat #10001D). ChIP’ed samples were treated overnight with proteinase K, followed by DNA purification with phenol/chloroform and ethanol precipitation, prior to downstream deep sequencing. For ChIP, GFP: Santa Cruz Cat #sc-9996, HA: Santa Cruz Cat #sc-805, custom rabbit anti-CENP-A #1 (epitope target: C-TPGPSRRGPSLGA), and mouse anti-S-tag antibody (Millipore Cat #MAC112) were used. ChIP experiments were reproduced from 2-5X. For complete TAU electrophoresis running procedure and membrane transfer parameters, please refer to [32] for Western/TAU protocols. Western detection: mouse anti-CENP-A (AbCam Cat #ab13939), mouse anti-S-tag (Millipore Cat #MAC112), biotinylated anti-GFP (AbCam Cat #ab6658), rabbit H1.5 (Invitrogen Cat #711,912 or rabbit H1.5 (AbCam Cat #ab18208) or custom rabbit, and guinea pig anti-CENP-C (MBL Cat #PD030) antibodies were used. Secondary antibodies from LiCor diluted to manufacturer’s specification and Western blots detected with LiCor M.

### Atomic force microscopy

Imaging of bulk, H3, CENP-A, and TAP-CENP-A chromatin was performed as described previously [33, 34] with the following modifications. Imaging was acquired by using commercial AFM equipment (Oxford Instruments, Asylum Research’s Cypher S AFAM) with silicon probes (OTESPA-R3 from Olympus with nominal resonances of ~ 300 kHz, stiffness of ~ 42 N/m) in noncontact tapping mode. Nucleosome dimensions were obtained and analyzed using ImageJ and graphs were prepared using ggplot2 package for R.

### Deep sequencing analysis

Paired raw reads were aligned to the Homo sapiens genome (version hg38) using bowtie2 (version 2.3.4.1). Final mapping results were processed using the HOMER suite [35] makeTagDirectory program to produce normalized tag density profiles. For each sample, peaks were called using the MACS2 (version 2.1.1.20160309) [36] callpeak function, using the sample’s respective input dataset, a q-value of 0.05 and the format BAMPE parameter. The replicate concordant peaks among replicates were combined to get the highly reproducible peaks using the IDR (Irreproducibility Discovery Rate) [37] method. 4462 narrow peaks for Native CpA and 1206 narrow peaks for CpA-TAP were identified by the MACS2. For the DAXX KO data set, 22,450 narrow peaks for Native CpA and 4,562 narrow peaks for CpA-TAP were produced after removing spurious peaks from Mock data by the bedtools intersectBed program (version 2.27.1). To identify genomic distribution by the replicate concordant narrowpeak files per condition, we used the University of California, Santa Cruz H. Sapiens hg38 annotated genes with the Homer suite program annotatePeak.pl. Heatmaps were generated over +/- 2 kb base pairs region around the center of the ChIPseq peak using an inhouse R package from the tag density profiles generated by the Homer suite. Heatmaps were sorted with in a group based in the average tag density of the site. Venn diagrams of shared overlapping peaks were produced using the Homer Suite mergePeaks, the R (version 3.5.2) and its package VennEuler. Karyoplots were generated using the R (version 3.5.2) and its karyoploteR and Bioconductor packages.

### CRISPR knock-in of CpA-TAP

Guide RNA design and selection: candidate guide RNAs targeting the C-terminus were designed using sgRNA Scorer 2.0 (PMID: 28,146,356) and subsequently tested for editing activity in 293T cells. Candidates 4150 (PAM sequence: GGGCCAGTTGCACATCCTTTGGG), 4154 (PAM sequence: AAGAGGATGAGCTTACCCCCTGG) were then selected as the guides for HDR experiments. Oligos encoding 4150, 4154 were phosphorylated and annealed and cloned into the pDG458 vector using a golden gate ligation reaction (PMID: 29,211,736). pDG458 was a gift from Paul Thomas (Addgene plasmid # 100,900 http://n2t.net/addgene:100900 ;RRID: Addgene_100900). Plasmids were sequence verified using Sanger sequencing.

Generation of donor construct: reference sequence for CENPA endogenous locus was downloaded from the UCSC Genome Browser (ENST00000335756.9). ~850 bp of sequence 5’ and 3’ of the stop codon was synthesized using Twist Biosciences and cloned into a minimal vector (pGMC00018; Addgene 195,320) with restriction enzymes in between to facilitate cloning of the TAP tag and P2A-Puromycin to generate an intermediate construct. P2A-Puromycin was then amplified from the Lenti-CRISPR-V2 vector (Addgene 52,961) and subsequently assembled into the intermediate construct. All cloning was done using Isothermal Assembly (PMID: 19,363,495). Plasmids were validated using Sanger sequencing.

## Results

### CpA-TAP has reduced affinity for CENP-C

**Fig. 1:**
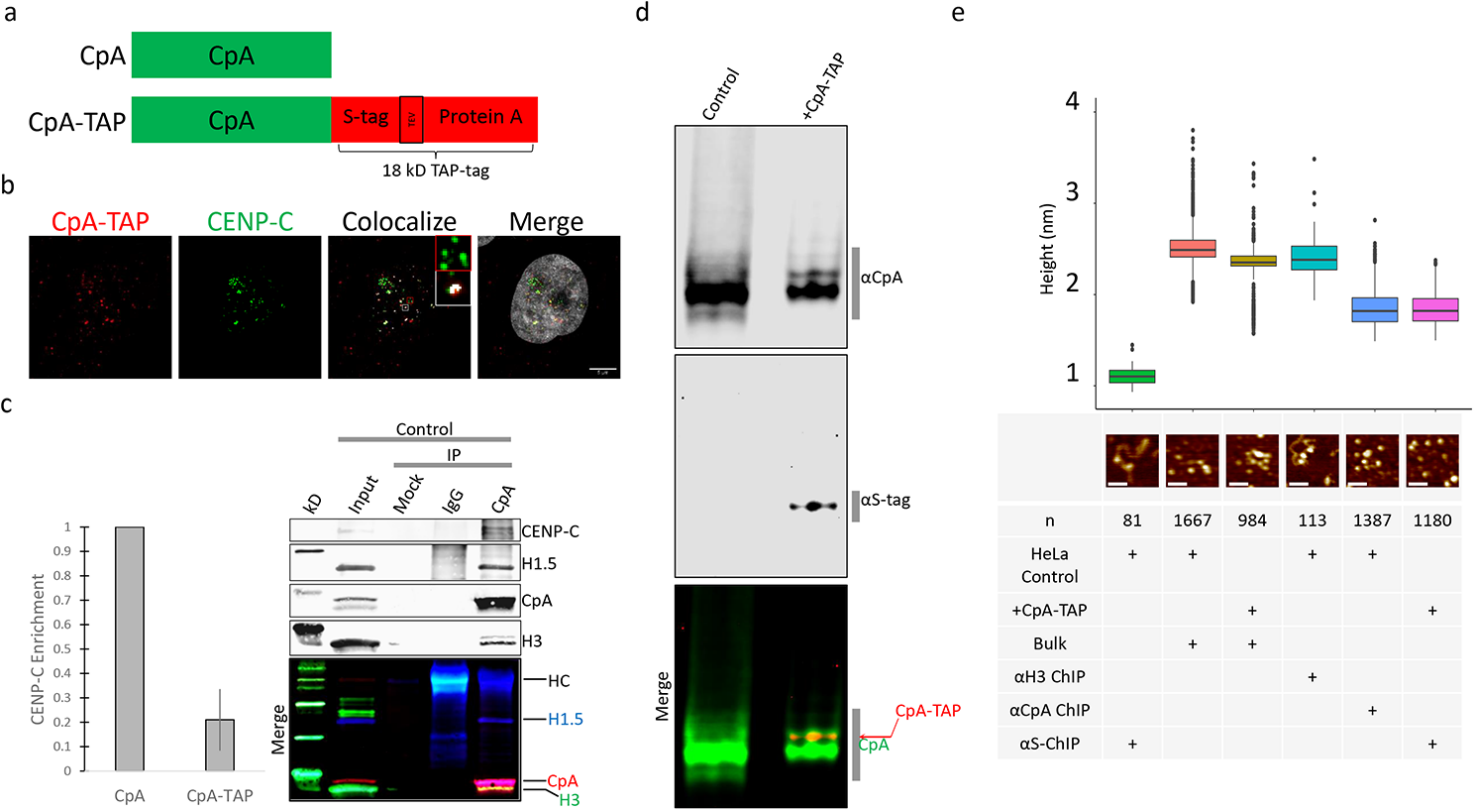
CpA-TAP has poor affinity for CENP-C and altered post-translational modifications (PTM) signature. **a**) Native CpA protein and fusion CpA-TAP protein consisting of CpA + S protein + TEV cleavage site + Protein A. **b**) CoIF of HeLa cells transiently expressing CpA-TAP with native CENP-C. Scale bar = 5 μm. **c**) Immuno-precipitation of native CpA versus CpA-TAP (S-tag IP) (see Fig. S1a) followed by Western detection for CENP-C, and quantification of the ratio of CENP-C enrichment normalized against CpA ChIP. Error bar represent SEM. HC = heavy chain. **d**) Long TAU (L-TAU) Western comparing control HeLa cells and cells with transiently transfected CpA-TAP (merged panel below). **e**) AFM heights for IP’ed native CpA versus CpA-TAP nucleosomes. AFM measurements were done in air mode. Bulk = extracted input chromatin and α-S ChIP = immuno-precipitated CpA-TAP chromatin. Scale bar = 50 nm

CpA-TAP consists of a C-terminal 18 kD modified Tandem Affinity Purification (TAP) tag that is made up of S-protein (one inactive component of ribonuclease S [38]-, a Tobacco Etch Virus (TEV) cleavage site (recognition peptide: E-N-LY-F-Q—S/G/A/M/C/H [39], and a minimal Staphylococcus aureus Protein A fragment with the calmodulin-binding peptide (10) (Fig. 1a). Stably expressed CpA-TAP chromatin was purified from human cells, and enriched with kinetochore components including CENP-B, CENP-H, CENP-N, CENP-T, and CENP-U when compared to H3.1-TAP chromatin [40]. These data support the interpretation that CpA-TAP successfully serves as a powerful biochemical tool to purify CENP-A associated complexes, which are otherwise present at low levels.

CENP-C directly interacts with CENP-A at the C-terminus [41, 42] and is required to bridge the connection between the centromere and kinetochore [43]. Interestingly, the TAP-tag is located at the C-terminal end of CENP-A. We, and others, have demonstrated the importance of the unstructured C-terminus of CENP-A in correctly recruiting, binding, and rigidifying CENP-A upon CENP-C binding [12, 15, 42, 44]. Indeed, swapping the C-terminus of CENP-A with that of histone H3 results not just in loss of CENP-C binding, but also in abrogation of CENP-A function [45]. Thus, we were curious whether the C-terminal tag impacts CENP-C binding, relative to native CENP-A.

First, we performed IF on transfected cells on control (untransfected) or transiently transfected + CpA-TAP HeLa cells, using anti-CENP-A or anti-S-protein (S-tag) antibodies, respectively. IF revealed poor colocalization between CpA-TAP with endogenous CENP-C (Fig. 1b).

Second, Chromatin Immuno-Precipitation (ChIP) followed by Western blots confirmed native CpA IP from control cells were enriched for CENP-C, but CENP-C was not enriched in the CpA-TAP (ChIP against S-tag protein) fraction (Fig. 1c and Fig. S1a). Similarly to Bailey et al. [46], we also observed that when CpA-TAP is co-expressed in HeLa cells, native CpA levels were reduced (Fig. S1a, b) and that CpA-TAP levels are four-fold reduced compared to native CpA (Fig. S1c). Native CpA was not co-purified with the S-tag IP, suggesting CpA-TAP does not completely occupy the same native CpA domains.

### CpA-TAP PTM signature is different from that of native CpA

Elucidating native CENP-A PTMs has been a challenging task, particularly due to its low abundance and difficulty in achieving complete peptide coverage during mass spectrometric.

(MS/MS) analyses. Previously, C-terminally FLAG-tagged CENP-A was immuno-precipitated and found to be ubiquitylated on lysine residue 124, which is important for centromeric deposition [24, 47]. More recent PTM analysis revealed a series of modifications that reside within the N-terminus of purified localization and affinity purification (LAP) tagged CENP-A, but modifications on CENP-A lysine residue 124 were absent [48]. How two CENP-A proteins with different tags can yield different PTM results remains unclear.

The use of alternative methods to successfully resolve different modified species of histones followed by mass spectrometry confirmation, have employed Triton Acid Urea (TAU) electrophoretic gel chemistry to successfully resolve proteins based on charge, hydrophobicity, and size [32, 49, 50] For example, we previously reported that native CENP-A from HeLa cells extracted with hydroxylapatite and high salt, followed by separation on a Long Triton Acid Urea (L-TAU) gel and analyzed by MS/MS, were acetylated on K124 [11, 12]. Other modifications at lower confidence levels were also detected along the N-terminus and throughout the histone fold domain (Bui, Nuccio, Nita-Lazar and Dalal, unpub). TAU gel electrophoresis remains a valuable qualitative tool to distinguish differing PTM signatures among two similar histones -in this case, native CpA versus CpA-TAP. The more CENP-A modified species exist, the greater number of bands or smears are expected on a L-TAU gel, as phosphorylated or acetylated residues cause protein bands to shift upwards [51]. Therefore, we used this method to compare CpA-TAP and native CpA purified from HeLa cells on L-TAU gels. Our results indicate that control HeLa cells exhibit at least four post-translationally modified forms of native CpA. To our surprise, only one distinct CpA-TAP species dominates and partially represses native CpA levels in + CpA-TAP transfected cells (Fig. 1d and Fig. S1d). These data suggest despite both proteins being CENP-A, the tagged version does not share the same PTM signature as native CpA.

### CpA-TAP and native CpA nucleosomes are indistinguishable in height

It has been previously reported that CENP-A nucleosomes undergo height transitions during replication [11]. We were curious whether adding a tag would alter nucleosomal heights in unsynchronized cells. Both native CENP-A and anti-S (for CpA-TAP) ChIP followed by Atomic Force Microscopy (AFM) measurements revealed that both types of nucleosomes were indistinguishable in height (Fig. 1e; Table 1), suggesting nucleosomal heights are dictated by the internal histone fold domain.

**Table 1:** AFM measurements of various nucleosomal structures

Nucleosome	*n*	Height (nm)	Diameter (nm)	Volume (nm^3^)
Untransfected (ChIP)	81	1.1 ± 0.1	13.2 ± 1.5	157 ± 46
Bulk (control)	1667	2.5 ± 0.2	14.9 ± 1.3	446 ± 106
Bulk (CpA-TAP)	984	2.3 ± 0.3	14.9 ± 2.2	420 ± 151
H3 (ChIP)	113	2.4 ± 0.3	13.5 ± 1.4	352 ± 91
CpA (ChIP)	1387	1.9 ± 0.2	13.9 ± 2.2	291 ± 112
CpA-TAP (ChIP)	1180	1.8 ± 0.2	14.8 ± 2.2	318 ± 126

### CpA-TAP deposition does not coincide with native CpA sites in the genome

In previous works, we, and others, have reported that CENP-A in certain cancer cells accumulates at ectopic or non-centromeric sites in the genome [31], and that this non-native pathway exploits H3.3 chaperones [52]. We observed that CpA-TAP is stably bound to chromatin but appears depleted for CENP-C (Fig. 1c). Therefore, we wanted to explore where CpA-TAP deposits in the genome. To achieve this, we performed either native CpA (in untransfected HeLa background) or S-tag (for CpA-TAP enrichment) ChIP, followed by deep sequencing (ChIPseq).

**Fig. 2:**
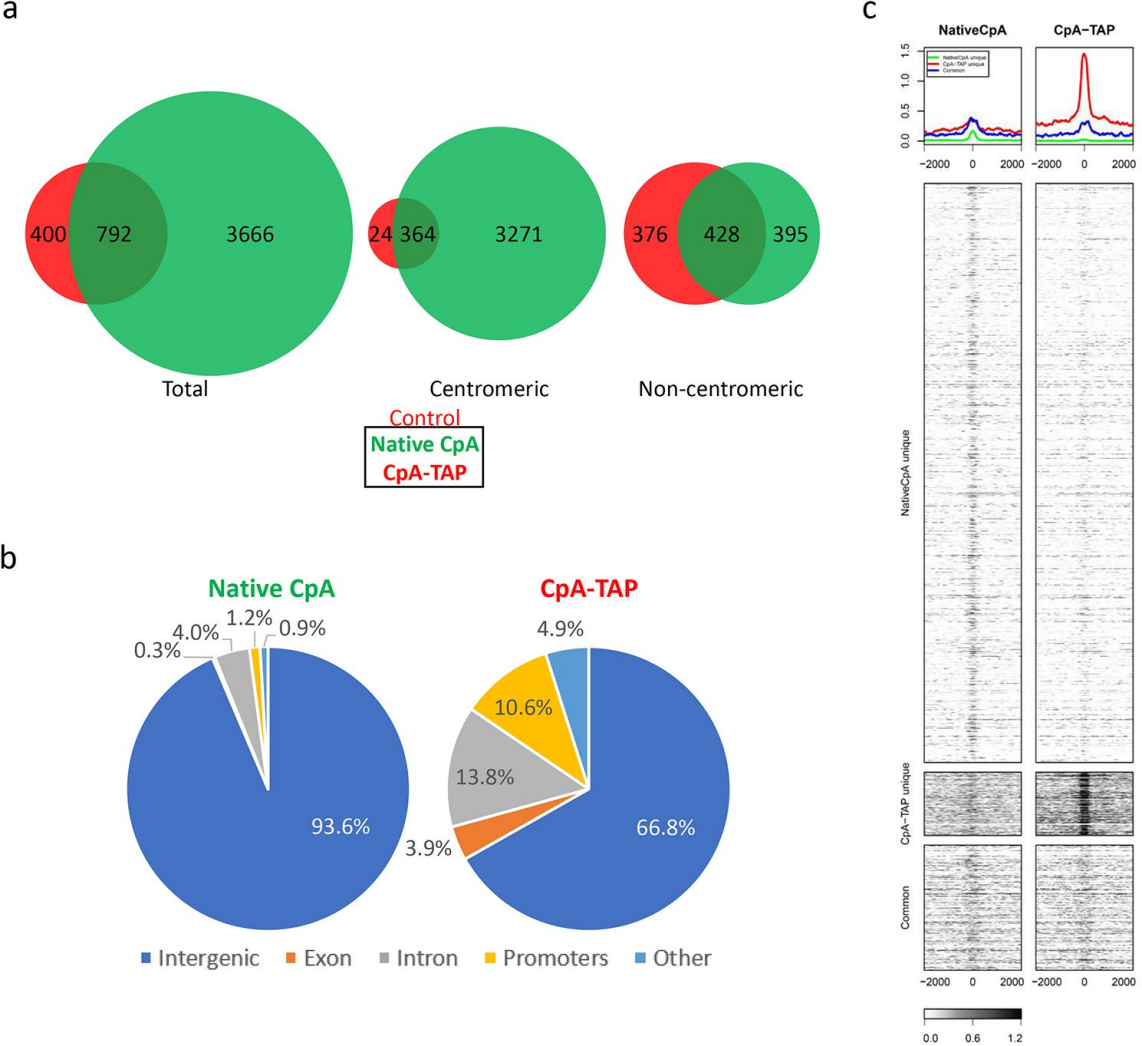
ChIPseq analysis of native CpA an CpA-TAP in control HeLa cells. **a**) Venn diagram depicting native CpA versus CpA-TAP total, centromeric, and non-centromeric hotspots. **b**) Categorical dissection of incorporated sites for native CpA versus CpA-TAP. **c**) Heat map of promoter occupancy for native CpA unique, CpA-TAP unique, and common sites

A total of 792 common hotspots between native CpA and CpA-TAP were identified, making up 18% of total native CpA and 66% of CpA-TAP (Fig. 2a). When the data was separated into centromeric versus non-centromeric identities, an interesting pattern emerged. Consistent with our prior analyses in HeLa cells (31), a vast majority of native CpA sites (82% (3,635/4,458)) in these cells are centromeric. In contrast, only 33% (388/1192) of CpA-TAP were enriched at centromeres. Thus, native CpA has the propensity for centromeric deposition greater than twice that of CpA-TAP (Fig. 2a). In the non-centromeric or ectopic fraction, both native CpA and CpA-TAP share more than 50% common hotspots (428/823 and 428/804, respectively). These data suggest that native and tagged CENP-A share more commonalities in their ectopic “off pathway” fraction than accurate HJURP-mediated deposition at centromeres.

When the hotspots are categorically separated into intergenic (sites not classified as either TSS, TTS, exon, 5’ UTR, 3’ UTR, and intron, but includes centromeres), exon, intron, promoters, and other types of domains, the differences between the two proteins are magnified. Native CpA makes up 93.6% of intergenic domains, while CpA-TAP only 66.8% (Fig. 2b). CpA-TAP makes up greater than 10-fold enrichment at exons (0.3% for native CpA versus 3.9% for CpA-TAP, respectively) and promoters (1.4% versus 10.6%, respectively), 5-fold enrichment at other/uncategorized domains (0.9% versus 4.9%, respectively), and 3-fold enrichment at introns (4.0% versus 13.8%, respectively) (Fig. 2b).

Global hotspot occupancy analysis reveals a stark contrast between native CpA and CpA-TAP. CpA-TAP occupancy at unique native CpA sites is lost, while CpA-TAP is enriched at sites that are specific to CpA-TAP (Fig. 2c). However, there are common sites between native CpA and CpA-TAP that remain unchanged (Fig. 2c).

We observed that CpA-TAP is stably bound to chromatin but depleted for CENP-C (Fig. 1c) and has a higher rate of deposition at non-centromeric domains (Fig. 2a-b). In previous works, we and others reported that CENP-A accumulates at non-centromeric sites in cancer cells [31, 53], and that this non-native pathway exploits the H3.3 chaperone, DAXX [52, 54, 55]. Therefore, we sought to determine whether CpA-TAP utilizes the DAXX-mediated pathway to deposit to these ectopic regions.

### DAXX promotes non-centromeric deposition of both native and tagged CpA

**Fig. 3:**
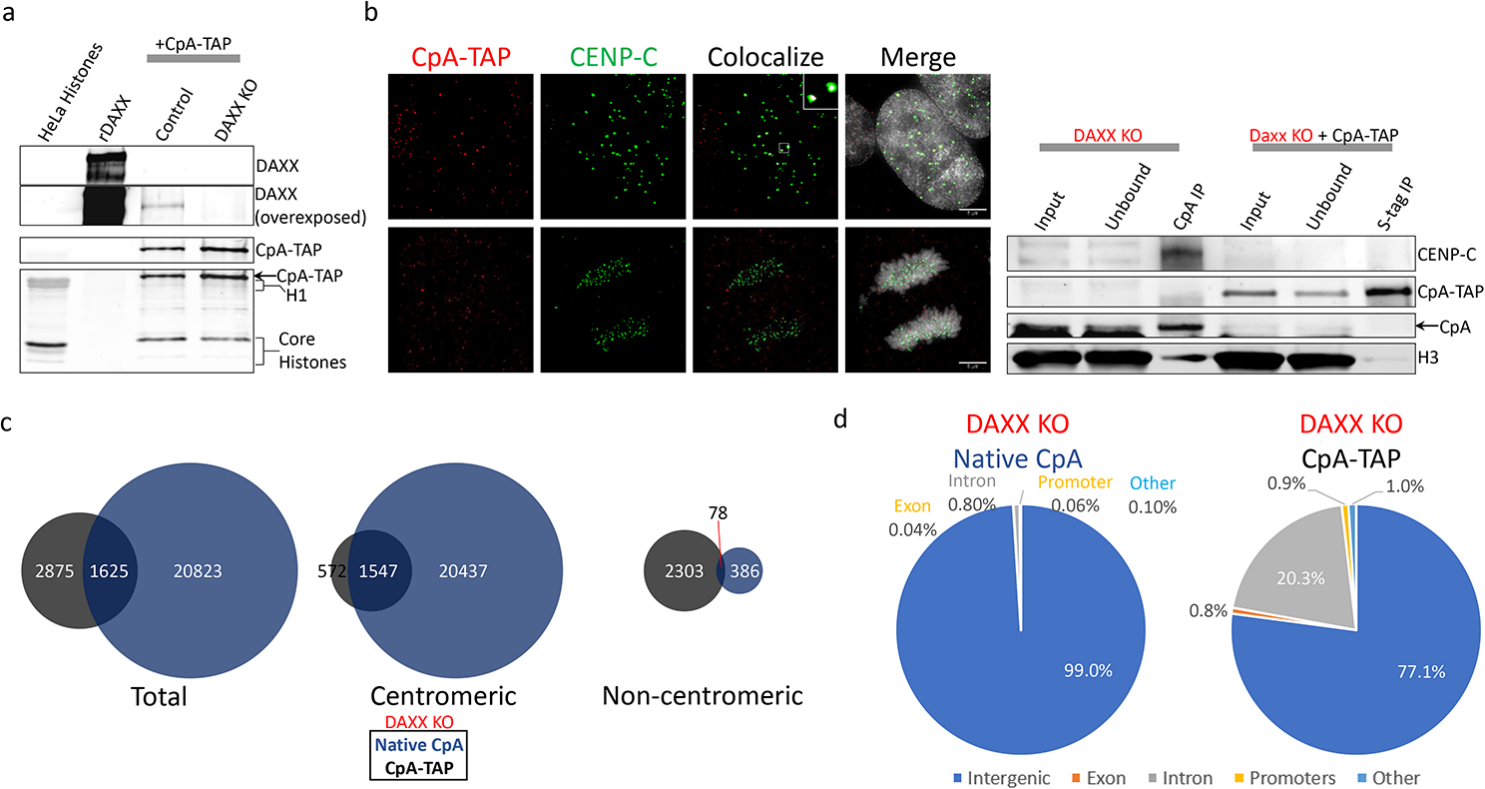
ChIPseq analysis of native CpA-TAP in DAXX KO HeLa cells. **a**) Western confirmation that DAXX is knock-out and that CpA-TAP is esxpressed (rDAXX: recombinant DAXX protein, AbCam cat #ab131785). **b**) CoIF of CpA-TAP and native CENP-C during interphase and mitosis (left panel), and native CpA versus S-tag ChIP followed by CENP-C Western (right panel). Scale bar = 5 μm. **c**) Venn diagram detailing total, centromeric, and non-centromeric hotspots for native CpA and CpA-TAP. **d**) Categorical dissection of native CpA versus CpA-TAP incorporated sites

Centromeric CENP-A relies on the chaperone HJURP to deposit at centromeres [20–22]. The predominant 67% (804/1192) non-centromeric deposition of CpA-TAP (Fig. 2a) led us to speculate that addition of the C-terminal TAP-tag can elicit the recruitment of an alternative chaperone. Previous reports suggest that ectopic CENP-A can be deposited by DAXX [52], and that its mis-localization is determined by the sensitive balance among chaperones HJURP, DAXX, and HIRA [54]. Westerns against S-tag (CpA-TAP) and DAXX were performed with HeLa histones (extracted with hydroxlyapatite and high salt), recombinant DAXX (rDAXX), and CpA-TAP transfected into both Control and DAXX KO cell lines confirmed DAXX was not expressed in the DAXX KO cell line (Fig. 3a).

In the DAXX KO cells, co-IF show partial colocalization between CpA-TAP and CENP-C on few centromeres outside of mitosis (Fig. 3b). Similarly, native CpA ChIP was enriched with CENP-C, while CpA-TAP did not pull-down detectable levels of CENP-C (Fig. 3b), consistent with the previous result that CpA-TAP has a lower affinity for CENP-C (Fig. 1c). To determine whether CpA-TAP utilizes DAXX as an alternative chaperone for the 67% sites that are non-centromeric (Fig. 2a), we performed a similar ChIPseq experiment after purifying native CpA or CpA-TAP but using the HeLa DAXX KO cell line. Native CpA (in the DAXX KO background) was further enriched at centromeres from 82% (in untransfected control) to 99% (21,984/22,448), while CpA-TAP at centromeres acquired a moderate increase from 32 to 47%.

(2,119/4,500) in the DAXX KO cell line (Fig. 3c). Noncentromeric deposition of native CpA decreased from 18% (823/4,458) to 2% (464/22,448) and CpA-TAP from 67% (804/1,192) to 53% (2,381/4,500) in the DAXX KO cell line (Fig. 3c), indicating DAXX plays a role in ectopic deposition of both native CpA and CpA-TAP.

Categorical dissection of the different occupied native CpA versus CpA-TAP sites in the DAXX KO cells add another intriguing layer of dynamics between the two. Native CpA now occupies 99% of intergenic sites (which includes centromeric regions) in the DAXX KO (Fig. 3d), compared to 93.6% (Fig. 2b). In the case of CpA-TAP, there is a 5- fold reduction from 3.9 to 0.8% at exons, and 10-fold reduction from 10.6 to 0.9% at promoters (Fig. 3c). One interesting point is that CpA-TAP at introns increased from 13.8% (Fig. 2b) to 20.3% (Fig. 3c) in the DAXX KO cell line, suggesting an alternative chaperone such as HIRA may be taking DAXX’s place, and that DAXX was repressing HIRA’s function at introns.

### Native CpA and CpA-TAP deposition at centromeres is enhanced and partially restored upon DAXX KO, respectively

**Fig. 4:**
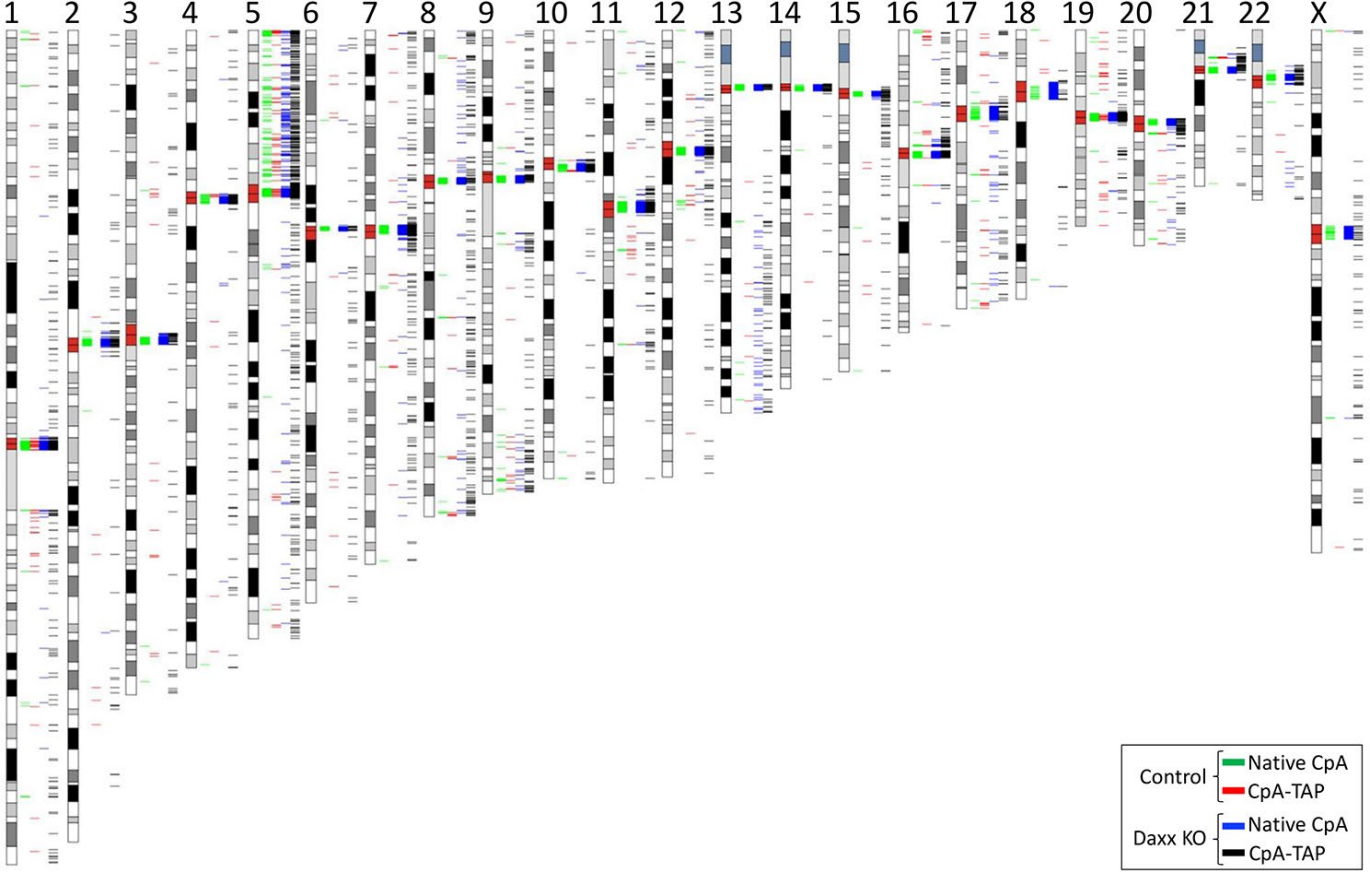
Karyoplot analysis of native CpA versus CpA-TAP deposition in both control (untransfected) HeLa and DAXX KO cells

With the exception of chromosome 5, native CpA (green) in control cells is predominantly centromere specific across all chromosomes (Fig. 4). However, CpA-TAP (red) is essentially void at centromeres on chromosomes 2–3, 6–18, 20–22, and X; moderately reduced at the centromere on chromosome 4; and mildly diminished on chromosomes 1, 5, and 19 in control cells when compared to native CpA (Fig. 4).

When DAXX is knocked-out, native CpA domains (blue) are noticeably enriched at centromeres on chromosomes such as chromosomes 17 and 18 (Fig. 4), suggesting DAXX knockout can lead to centromeric expansion. The most fascinating observation is that CpA-TAP returns to centromeres on all chromosomes in the DAXX KO cell line (Fig. 4). The DAXX KO cell line revealed that though both native CpA and CpA-TAP are enriched at centromeres, knocking out DAXX may simultaneously increase ectopic deposition for both proteins on most chromosomes (Fig. 4).

### Introducing tagged CpA disrupts and redistributes native CpA in control HeLa and DAXX KO cells

**Fig. 5:**
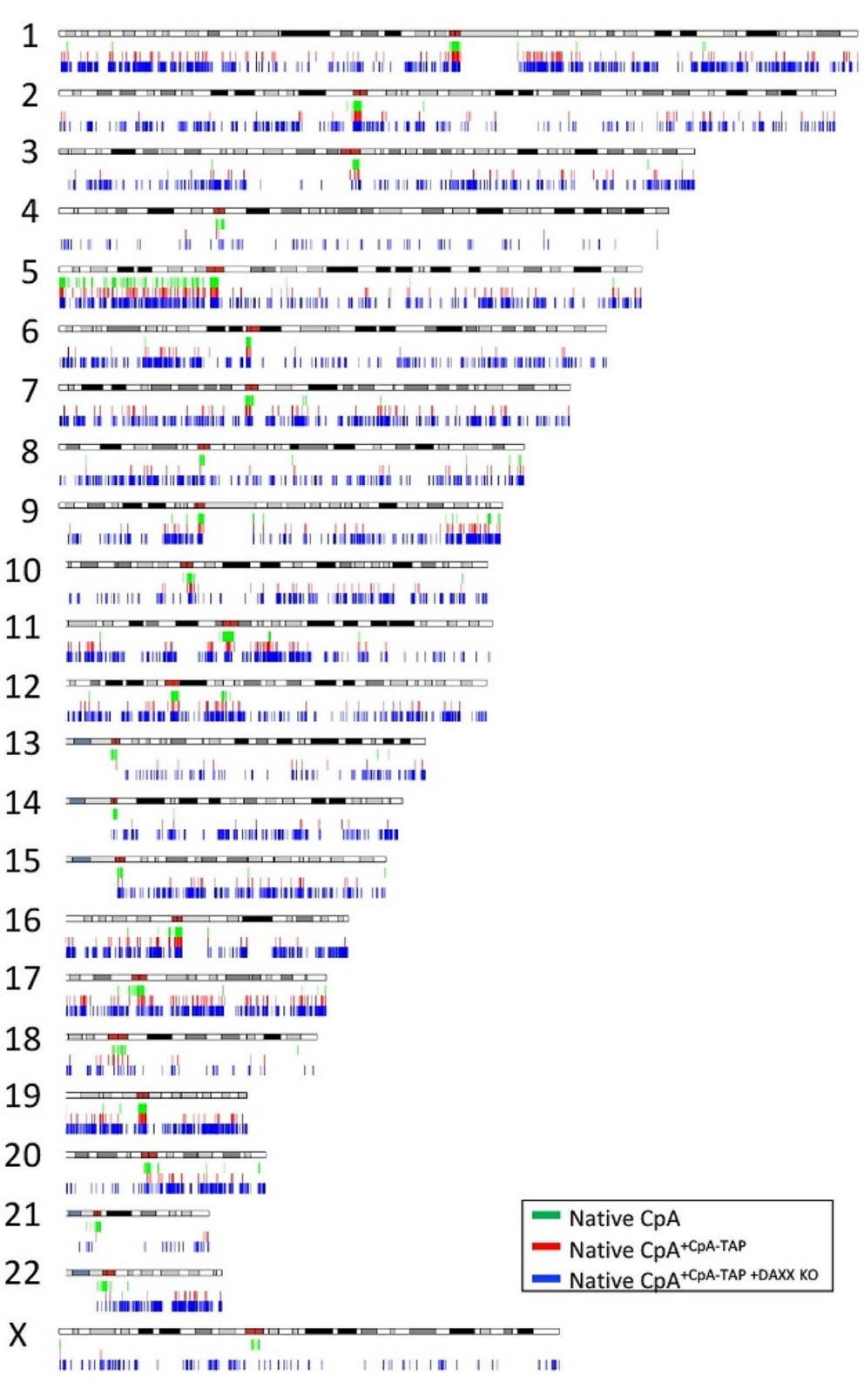
Karyoplot analysis of native CpA in control HeLa cells, versus in the presence of CpA-TAP, and versus in the presence of CpA-TAP + DAXX KO.

It is not known whether introduction of foreign CpA-TAP can disrupt native CpA deposition within the genome. To ascertain the impact (if any), we transfected CpA-TAP to both HeLa and HeLa cells where DAXX is knocked out, followed by first serial depletion of CpA-TAP with S-tag ChIP and then native CpA ChIP. Much to our surprise, CpA-TAP introduction led to shrinkage of the native CpA centromeric domain with simultaneous expansion of the ectopic domains (red native CpA^+ CpA−TAP^) when compared to native CpA (green) under control HeLa conditions (Fig. 5). Centromeric deposition of native CpA was either gained or loss depending on the chromosome (blue), but non-centromeric domains were significantly expanded when CpA-TAP was introduced and DAXX was knocked-out (blue native CpA^+ CpA−TAP +DAXX KO^) compared to native CpA (green) (Fig. 5). A summary of observations for native CpA versus CpA-TAP among various conditions can be found in Table 2.

**Table 2:** Assessing centromeric versus non-centromeric deposition of native CpA versus CpA-TAP under various conditions. + to +++ : range of deposition levels; - : no deposition observed; N/A : not applicable; … : range of varying degrees of deposition, depending on chromosome

	Native CpA		CpA-TAP	
	Centromere	Non-centromere	Centromere	Non-centromere
Control HeLa	+++	-	N/A	N/A
+DAXX KO	+++ … ++++	+	N/A	N/A
+CpA-TAP	+ … ++	++	- … +	+
+DAXX KO +CpA-TAP	- … +	+++	++ … +++	+++

### CRISPR knock-in of CpA-TAP does not recapitulate native CpA function

To determine whether CpA-TAP can fully replace native CpA in vivo, we performed a knock-in of the C-terminal TAP-tag to the endogenous CpA locus in both control HeLa and HeLa cells where DAXX has been knocked out. Six colonies from each cell line were isolated and expanded for further downstream applications. In the case of HeLa control cells, CpA-TAP knock-in led to all six colonies not surviving past 7 days (Table 3). For HeLa cells where DAXX was knocked out, only colonies #2, 4, 5, and 6 survived, continued to divide (Table 3), and were confirmed heterozygous (Fig. S2a). Colonies #1 and #3 failed to divide and perished after 1 month.

**Table 3:** CRISPR knock-in of CpA-TAP to recapitulate native CpA function. - : non-viable colonies; + : colonies that grew and maintained under puromycin selection

	#1	#2	#3	#4	#5	#6
Control HeLa	**-**	-	-	-	-	-
DAXX KO	-	+	-	+	+	+

After 5 months, the same heterozygous colonies in the DAXX KO background were assessed for CpA-TAP protein expression, and intriguingly, all colonies that formerly expressed CpA-TAP no longer did (Fig. S2a-b), suggesting cells were preferentially expressing native CpA while silencing CpA-TAP.

### N-terminal SNAP-CpA has reduced *de novo* early G1 phase deposition

HJURP-dependent CENP-A deposition at centromeres occurs during G1 phase (20, 21). To determine whether a tagged version of CENP-A impacts deposition during early G1 phase, we utilized a similar approach to Jansen, et al. but cloned an N-terminally SNAP-tag CENP-A downstream to the CMV promoter [56]. The transiently transfected cells were synchronized with a double thymidine block, pulse-chase labeled with TMR Star (red), and coIF with anti- native CpA (green). Though we typically observe > 90% transfection efficiency with electroporation, among the 200 cells with native CpA IF centromeric signals observed, < 50% of those SNAP-CpA containing cells showed colocalization with native CpA (co-IF with native CpA in green) (Fig. S3 and S4).

### Native CpA, CpA-TAP, and GFP-CpA vary in centromeric deposition

**Fig. 6:**
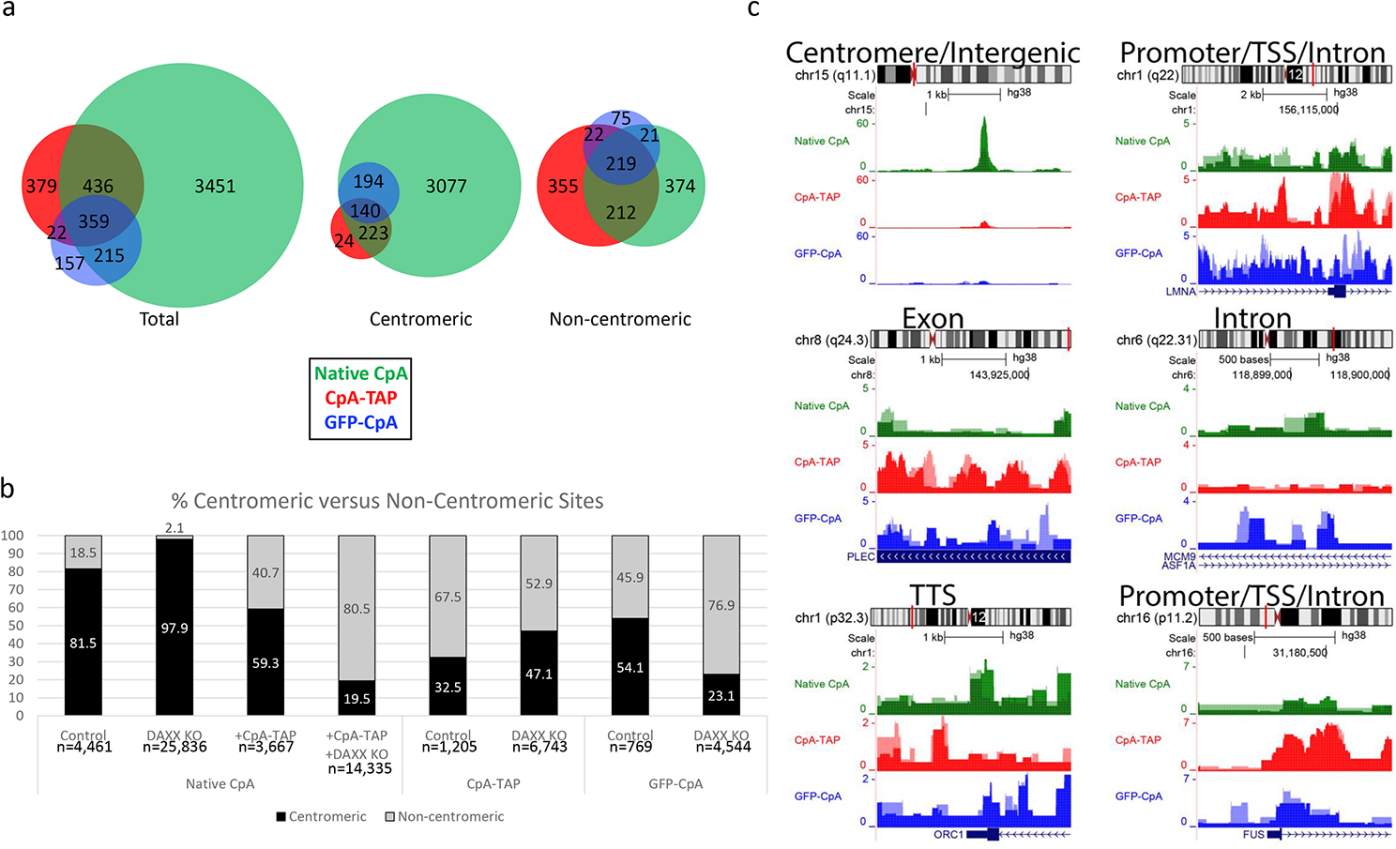
Native CpA have differing deposition profiles compared to N-terminally tagged GFP-CpA and C-terminally tagged TAP. **a**) Triple Venn diagram highlighting overlapping and non-overlapping sites among native CpA, CpA-TAP, and GFP-CpA. **b**) Percentage of centromeric versus non-centromeric sites of native CpA, CpA-TAP, and GFP-CpA under various treatments. **c**) Peak snapshots of several genic regions for native CpA, CpA-TAP, and GFP-CpA

Much of the study thus far has relied on a single C-terminally tagged CpA-TAP construct. We were curious whether we would observe similar differences if we were to utilize an N-terminally.

tagged GFP-CpA, which has been previously reported in IF studies [11, 12]. GFP-CpA is 40 kD, slightly larger than CpA-TAP (Fig. S1). While total sites detected by GFP-CpA are fewer than native CpA and CpA-TAP, all its centromeric sites coincide with native CpA and the majority of its non-centromeric sites are shared between native CpA and/or CpA-TAP (Fig. 6a). When examining each protein’s centromeric affinity, native CpA tops at 81%, followed by GFP-CpA (54%), and CpA-TAP (32%) (Fig. 6b).

Further inspection of the different loci provides details of how the three proteins behave. For instance, at the centromere and one of the TTS, native CpA is significantly enriched while CpA-TAP and GFP-CpA are nearly void (Fig. 6c). On the flip side, CpA-TAP and GFP-CpA (to a lesser degree) have higher affinity for certain exons, promoters, and TSS compared to native CpA (Fig. 6c).

### Tagging histone variants disrupt nucleosomal protein-protein interactions

**Fig. 7:**
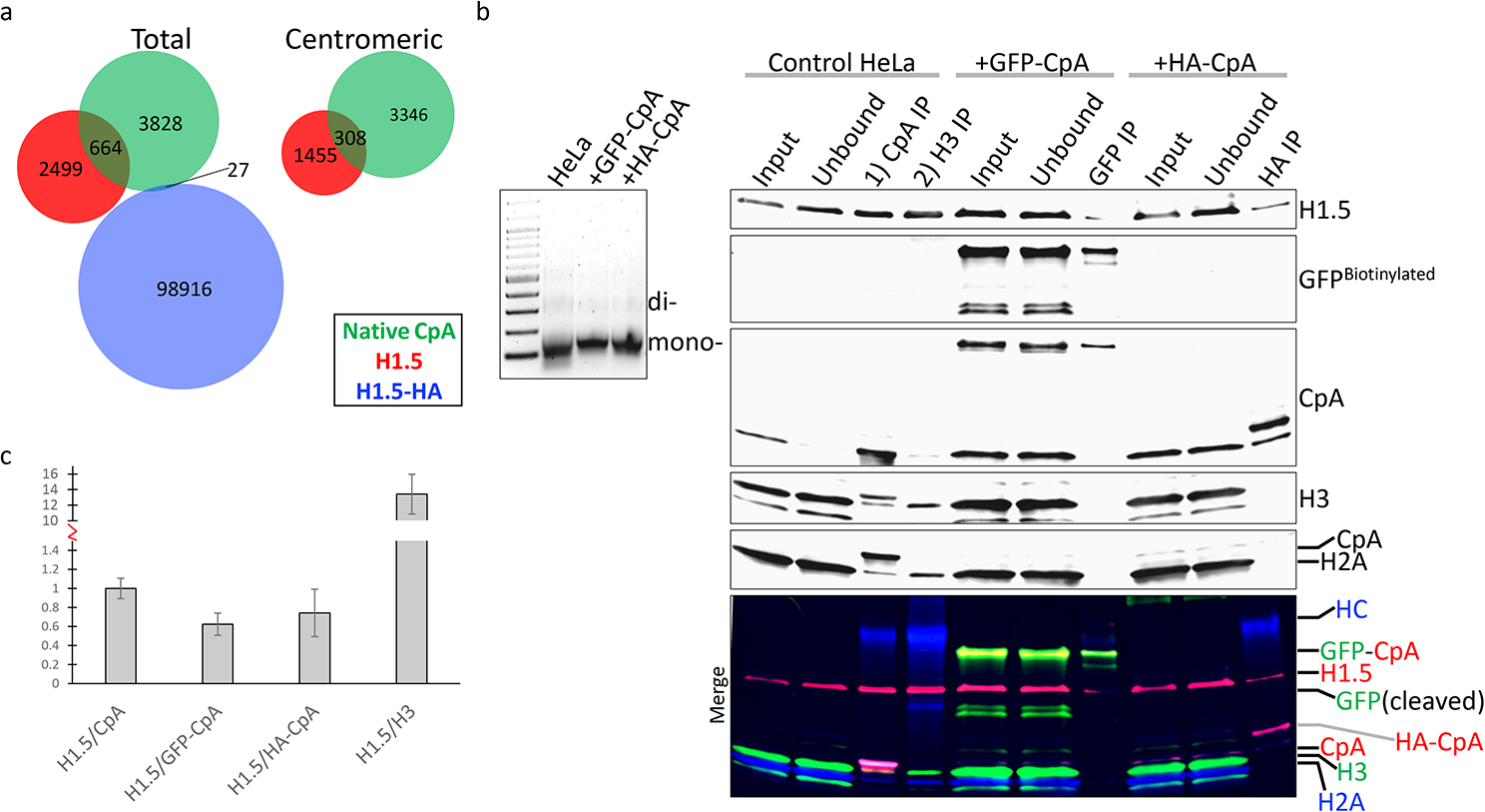
Tagged and untagged histone variants differ in genome wide distribution and nucleosomal interactions. **a**) Previously reported H1.5 and H1.5-HA ChIPseq sites were compared to native CpA ChIPseq sites from this study. **b**) ChIP performed against native CpA, GFP-CpA, and HA-CpA mono-nucleosomes and probed for histone H1.5 (Invitrogen Cat #711,912). HC = heavy chain. **c**) Ratios of H1.5/native or tagged CpA, normalized against H1.5/native CpA, from 2–5 independent experiments. Error bars = SEM

It is not known whether introduction of a tagged histone variant can impact protein-protein interactions within the nucleosomal context. Of the several available native versus untagged ChIPseq datasets for histone variants, linker histone H1.5 was readily available [57, 58] and its interaction with CENP-A previously deemed unlikely [59]. To determine whether H1.5 (GSM5076929) and tagged H1.5-HA (GSM1197474) histones had altered genomic deposition, we turned to previously reported ChIPseq results [57, 58] and reanalyzed both sequencing datasets for total and centromeric sites. Out of the total number of sites for native H1.5, 664/3,163 (21%) colocalized with total native CpA (Fig. 7a); Whereas 27/98,943 (0.03%) H1.5-HA sites colocalized with total native CpA (Fig. 7a). When we narrowed down our search to overlapping centromeric sites with native CpA, 308/1,763 (17%) of native H1.5 are found with native CpA; However, no centromeric and overlapping native CpA sites were found when examining H1.5-HA (Fig. 7a).

Previously, it was reported that epitope tagged CENP-A failed to interact with histone H1.5 [59].We hypothesized that tagging CENP-A could disrupt the H1.5 interaction. To test this, we performed native CpA ChIP on mono-nucleosomes from untransfected control HeLa versus GFP-CpA and HA-CpA ChIP in transfected HeLa cells, followed by Western analysis. Similar to previous results when adding a TAP-tag to CpA disrupts CENP-C binding (Fig. 1c), our data indicates tagging CpA may disrupt histone H1.5 interactions, depending on the type of engineered tag utilized (Fig. 7b-c and Fig. S3a-c). Sequential native CpA followed by native H3 ChIP revealed H3 binds H1.5 with a ~ 13-fold higher affinity than native CpA (Fig. 7c).

## Discussion

The use of engineered tags has provided mechanistic insights and led to many valuable discoveries, making their use ubiquitous in molecular biology. Whether it is short with several amino acids or a truncated protein, not all tags are created equal, and each has its own purpose.

Unlike canonical histones, histone variant CENP-A is found at significantly lower levels, making it a challenge to purify and investigate. Thus, adding a tag often eliminates this challenge and eases downstream applications. One commonly reported tagged CENP-A is CpA-TAP. In our studies, IF data indicate that CpA-TAP and CENP-C poorly co-localizes (Fig. 1b). Similarly, ChIP followed by Westerns reveal robust enrichment of CENP-C with native CpA, but not with CpA-TAP (Fig. 1c and Fig. S1a). One logical explanation is that the large 18 kD C-terminal tag reduces CENP-C’s accessibility to CENP-A’s C-terminal tail. The C-terminal domain is necessary for CENP-C recognition and stabilization, and rigidification of CENP-A nucleosomes at the centromere [41, 45]. Both IF and Western data suggest that addition of the TAP-tag interferes with CENP-C binding, and when given a choice, cells prefer depositing native CpA, instead of CpA-TAP, at centromeres.

We note some caveats, that led us to treat our results with caution. First, our study is done using transiently transfected cells, harvested 3 days post-transfection, resulting in cells that express CpA-TAP at a significantly reduced fraction compared to native CpA. This is in contrast to the previous study [40] when stable cells selected for high CpA-TAP levels relative to low native CpA, showing CpA-TAP (after TEV cleavage) co-IP’s with CENP-C. One explanation is high CpA-TAP levels compensate for insufficient native CpA levels, allowing CpA-TAP to successfully compete for HJURP recognition. However, in a competitive environment where native CpA levels are relatively high compared to transiently expressed CpA-TAP, HJURP still retains a preference for native CpA to deposit at centromeres.

In addition to transient transfection, we sought to knock-in CpA-TAP to determine whether it is capable of recapitulating loss of native CpA. The inability to sustain colonies containing CpA-TAP in control HeLa beyond a week post-transfection and to identify homozygous CpA-TAP colonies in DAXX knock-out HeLa cells indicate CpA-TAP cannot fully replace native CpA and thus, are not functionally equivalent (Table 3).

Given these observations, a logical extension is that chromatin remodelers and modifiers might find CpA-TAP a poor structural substrate for modification, if nucleosomal access, location, or stability is hindered by the tag. We were curious whether CpA-TAP undergoes PTMs that differ compared to native CpA. Using the TAU electrophoretic gel chemistry that separates proteins primarily based on charge [49, 51], we were able to distinguish disparate PTM migration species between native CpA versus CpA-TAP. Our data reveals a single modified CpA-TAP species, while native CpA has at least four distinct modified species (Fig. 1d). However, it must be pointed out that CpA-TAP protein levels are 4-fold lower than native CpA (Fig. S1c), so it may be plausible that minor modified CpA-TAP proteins are below detectable limits for TAU gel electrophoresis. Because no structural data for CpA-TAP nucleosomes exist, we cannot be certain whether the histone-sized tag interferes with the modifying enzyme’s accessibility to CpA-TAP.

How a tag can influence a protein’s ability to interact with other proteins and have its PTMs altered is unknown. However, we can speculate that the tags may interfere with certain interactions depending on whether it is N- or C-terminally tagged. A C-terminal TAP tag is expected to have more impactful structural implications as the CENP-A C-terminal end is essential for kinetochore CENP-C binding [41, 42], which we show is severely impacted for CpA-TAP (Fig. 1c). When a larger GFP tag is added on the N-terminus, 54.1% of GFP-CpA are centromeric, which is more than CpA-TAP (32.5%) but lower than native CpA (81.5%) (Fig. 6b), suggesting location of the tag can dictate behavior and deposition.

Because CpA-TAP does not completely co-localize with CENP-C, we surmised that it may be deposited at ectopic domains, which we verified by ChIPseq. Surprisingly, 67% of CpA-TAP hotspots are non-centromeric, while 82% of native CpA hotspots are centromeric (Fig. 6b). Delimitating which genomic category each protein occupies, we observed native CpA coalesces at intergenic regions (which includes centromeres) for 93.6% versus CpA-TAP only 66.8% (Fig. 2b). CpA-TAP invades other genic regions including exons, introns, and promoters several fold more than native CpA (Fig. 2b). Upon further examination, the centromere of chromosome 15 is enriched with native CpA but not CpATAP. Collectively, the data implicates a chaperone other than (centromere-specific) HJURP is responsible for the ectopic deposition of CpA-TAP, likely DAXX or HIRA.

Previous studies reported that DAXX ectopically deposits CENP-A [52, 54] to regions outside the centromere, so we expressed CpA-TAP in a HeLa cell line where DAXX was knocked-out (Fig. 3a). IF and ChIP followed by Western confirm CpA-TAP still poorly colocalizes with CENP-C in the DAXX KO cell line (Fig. 3b). ChIP followed by deep sequencing revealed the number of native CpA centromeric sites increased from 82 to 98% (21,984/22,448), and the number of CpA-TAP centromeric sites also mildly increased in Control versus HeLa DAXX KO cell lines, respectively (Fig. 3c). Data from both cell lines implicate DAXX as a driving force for ectopic deposition of both native CpA and CpA-TAP.

Categorical dissection of the different domains that native CpA and CpA-TAP occupy in the DAXX KO cell line reveals another chaperone may contribute to CpA-TAP’s ectopic deposition. While native CpA occupation at exons, introns, and promoters were all reduced, CpA-TAP occupation was only reduced at exons and promoters in the DAXX KO cells when compared to control HeLa cells (Fig. 3d). CpA-TAP occupation at introns increased from 13.8 to 20.3%, in control HeLa versus DAXX KO cells, respectively (Fig. 3d). This evidence suggests that a third chaperone, possibly HIRA, can compete with DAXX to chaperone CpA-TAP to introns,

especially once DAXX is removed.

Karyotypic analyses done on native CpA and CpA-TAP under various cell lineages or treatments provide valuable insight into how these two histone variant proteins localize or deposit in the genome. Though native CpA deposits predominantly to centromeres across all chromosomes, CpA-TAP deposits at centromeres only at a few chromosomes -the rest are non-centromeric (Fig. 4). Only when DAXX is knocked-out, does CpA-TAP return to centromeres, suggesting DAXX prevented centromeric deposition of CpA-TAP (Fig. 4). Interestingly, upon DAXX knock-out, non-centromeric deposition of CpA-TAP became more evident in several chromosomes, similarly to native CpA (Fig. 4). Taken together, DAXX repression allowed for simultaneous enrichment of native CpA and CpA-TAP both at centromeres AND non-centromeres on several chromosomes, suggesting DAXX may play an important role as ‘balancer’ to reassign both proteins to either HJURP or HIRA for deposition. Only when the ‘balancer’ is removed does a chaperone free-for-all (HJURP and HIRA) ensue to determine where the protein is deposited.

The more disturbing observation is that upon introduction of CpA-TAP to cells, several native centromeric CpA sites are displaced and undergo altered deposition to non-centromeric regions (Fig. 5). The non-centromeric deposition becomes even worse for many chromosomes when DAXX is knocked out (Fig. 5), suggesting there is a complicated relationship between CpA-TAP introduction and chaperone maintenance. The data implies introducing a foreign tagged histone variant can have dramatic and unforeseen impact on both the tagged and native histone variant as well.

How the addition and even location of a tag can alter chaperone dynamics and function of CENP-A is an open question. It was previously determined that when yeast CENP-A (Cse4) is either internally or C-terminally tagged with GFP, the result is either normal functionality or delayed growth, lethality at higher temperatures, and accumulation at ectopic sites, respectively [60]. Remarkably, when we examined N-terminally tagged GFP-CpA, we observed that GFP-CpA had a higher affinity for centromeres than C-terminally tagged CpA-TAP, but still not as much as native CpA (Fig. 6b), consistent with the previous study that observed hyper-accumulation of C-terminally tagged CpA at ectopic sites [60]. This would imply that positioning of a tag on a histone variant can alter spatial and functional outcomes.

Another conundrum is when HeLa cells were transfected with GFP-CpA, a portion of the tagged protein was cleaved, resulting in cleaved GFP in both input and unbound fractions that are resistant to anti-GFP immuno-precipitation (Fig. 7b). This implies the GFP-tag is mis-folded and its epitope hidden from antibody detection during ChIP. How cells can distinguish and cleave off the tag is unknown.

Global examination and classification of the native versus tagged CpA proteins reveal complex dynamics and interactions. For example, when DAXX is knocked-out, native CpA deposition at centromeres is increased. However, when DAXX is knocked-out in the background of CpA-TAP introduction, native CpA deposition at centromeres is reduced by 75% (Fig. 6b), lending further support that introducing a foreign tagged histone variant can have unanticipated global genomic implications.

All the data thus far led us to look back to previous studies that inferred native CENP-A dynamics using tagged CENP-A as the readout. One study concluded that epitope tagged HA-FLAG-CENP-A when immuno-precipitated, did not interact with linker histone H1 in vivo [59]. We speculated that addition of the tag may disrupt native CENP-A’s ability to (1) interact with histone H1s, particularly histone H1.5 and (2) form stable H2A-containing nucleosomes. To address this, we performed native (unfixed) ChIPs from mono-nucleosomes against native CpA, GFP-CpA, and HA-CpA, followed by Westerns against H1.5 using three antibody sources (one custom and two commercial) (Fig. 7b and S3). In all cases, native CpA ChIP was enriched for histone H1.5, HA-CpA to a lesser degree, and GFP-CpA poorly associated with H1.5 (Fig. 7b-c and S3). Additionally, we were surprised to see that the immuno-precipitated tagged CpA proteins had significantly reduced levels of histone H2A, suggesting the tagged CpA histones are less likely to form functional H2A-containing nucleosomes (Fig. 7b). Our data supports the conclusion that epitope tagged CENP-A does disrupt histone H1 binding as previously reported [59], but that it also does not fully recapitulate native biological interactions and forms fewer H2A-containing nucleosomes in vivo. An alternative explanation is that many of these tagged CpAs are transiently deposited but not stably incorporated as H2A-containing nucleosomes. This would explain how GFP-CpA is known to be strongly associated with centromeres during IF, but only 54% are classified as centromeric during ChIPseq analysis. Transient deposition does not necessarily equate to stable nucleosomal incorporation and kinetochore protein CENP-C interaction.

Another surprising observation is when re-examining and comparing previously deposited ChIPseq data for native H1.5 versus H1.5-HA [57, 58], our analysis revealed the two proteins are very different. There was no genomic overlap between H1.5 and H1.5-HA, and though 17% of native H1.5 occupy centromeres, 0% of H1.5-HA are found at centromeres (Fig. 7a). This observation led us to conclude that addition of tags to other histone variants (besides CENP-A) may lead to unknown consequences such as not fully recapitulating biological functions of the native protein in vivo. A recent report suggest that the non-histone tagged protein Lamin A, essential for nuclear function and morphology, when tagged, has impaired structural support [61]. These examples of histones and non-histone proteins having altered function when tagged suggest undetermined functional outcomes of tagging proteins may be more prevalent than previously thought.

What is perhaps striking from the cancer biology perspective is that the chimeric nature of these tagged CpA-fusion proteins bears resemblance to fusion proteins that are characteristic cancer markers, which often result in low tissue specificity [62]. Under high levels of genome instability, transcript fusions which are often a result of chromosomal rearrangements, can lead to expression of aberrant proteins as documented with in-frame protein kinases found in bladder carcinoma, glioblastoma, lung adenocarcinoma, etc [63]. Additionally, a fusion protein’s capability to hijack an alternative chaperone has previously been reported. Fusion of FMS-like tyrosine kinase-3 (FLT3) with the HLH-transcription factor TEL (TEL/FLT3) forms dimers and is mediated by chaperone GRP94 [64]. However, fusion with ETS variant transcription factor 6 (ETV6) produces an ETV6/FLT3 oncoprotein fusion that is constitutively active and utilizes Hsp90, a chaperone known to stabilize a number of proteins required for tumor progression [65]. Interestingly, Hsp90 is necessary for ubiquitylation of CENP-A K124 and centromeric deposition of CENP-A-FLAG [66]. Here, we show for the first time, how fusion of CENP-A to a histone-sized TAP-tag can alter histone chaperone dynamics, resulting in what is normally and predominantly centromeric deposition via HJURP, to ectopic deposition via DAXX, and speculate that the aberrant nature of tagged CpA resembles transcript fusions serving as cancer biomarkers.

## Conclusions

Though epitope tags remain instrumental in the quest for elucidating countless pathways and scientific processes that would otherwise remain unknown, their use should be properly controlled, and its users cautious. Our data suggests that the assumption that tagged histone variants are functionally equivalent to their native or endogenous untagged counterparts, can no longer be made. Experimental controls dictate H3-TAP is a proper and sufficient control for CpA-TAP, but confirmation that the fusion CpA-TAP protein can fully recapitulate biochemical, cytological, biophysical, and genetic/genomic characteristics of native CpA would be most ideal. Upregulated CENP-A has been documented to lead to genome instability [67], found in cancer [68], and often leads to ectopic deposition in cancer cells [31]. Understanding how CpA-TAP utilizes an alternative chaperone to deposit at ectopic sites can provide valuable insight into histone turnover and deposition during tumorigenesis. Though the use of tags and resulting fusion proteins to exploit non-native cancer pathways is a surprising finding and its mechanism is not fully understood, the use of tags offers another valuable tool to further elucidate non-native cancer pathways.

### Electronic supplementary material

Below is the link to the electronic supplementary material.

Supplementary Material 1

## Data Availability

Data generated and analyzed in this study are included in this published article and the raw ChIP-seq data (i.e., fastq files) and MACS2 narrow peak files have been deposited at NCBI GEO (GSE209933).
