# Supplementary material for: Native and tagged CENP-A histones are functionally inequivalent
Source: Epigenetics Chromatin. 2024 Jun 2;17:19. doi: 10.1186/s13072-024-00543-9 (PMC11145777; doi:10.1186/s13072-024-00543-9)
Supplement: Supplementary file 1 — Supplementary Material 1 [file 13072_2024_543_MOESM1_ESM.docx]

Fig. S1: Biochemical differenc
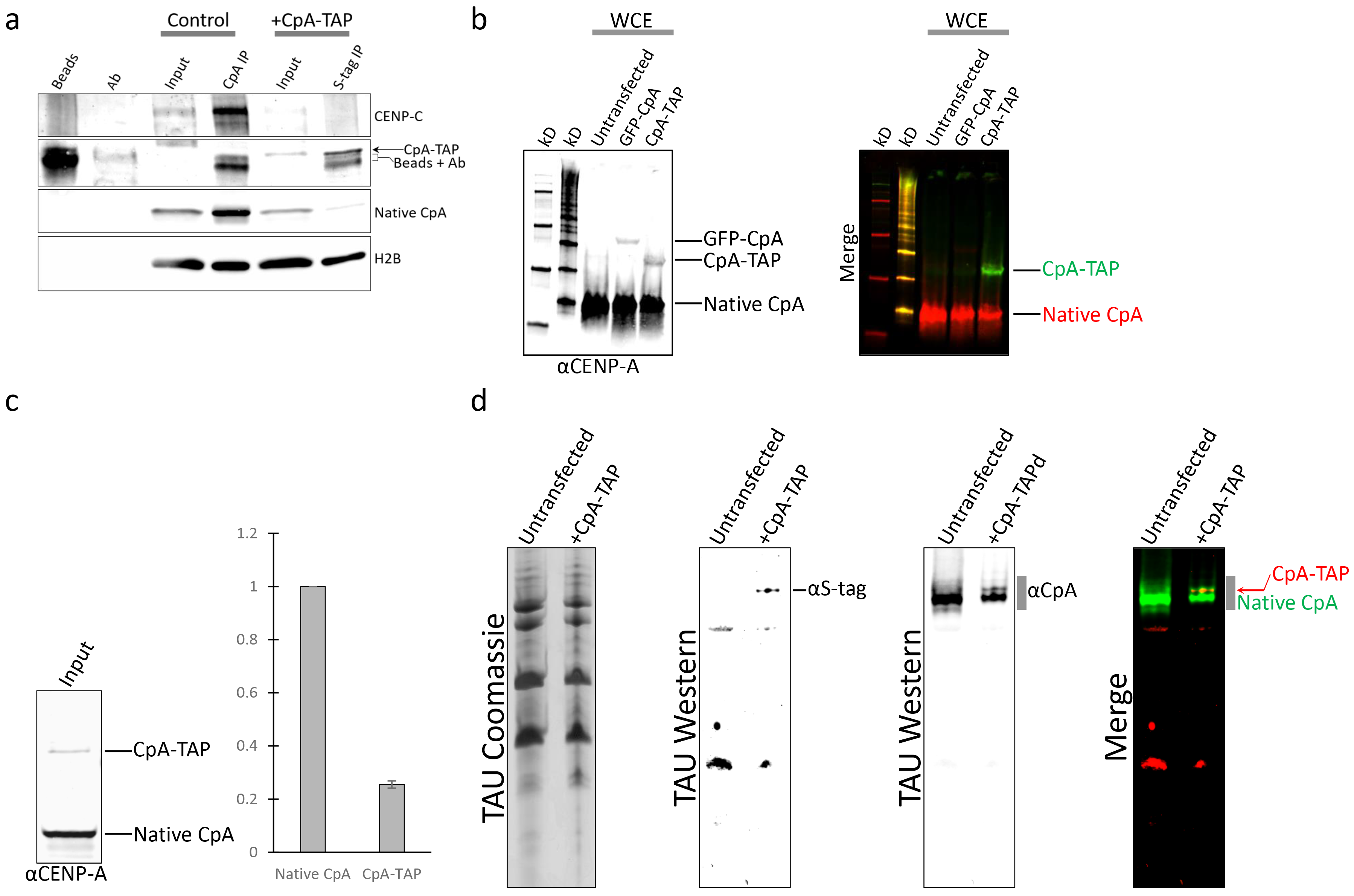
es between native CpA, CpA-TAP, and GFP-CpA. a) ChIP for native CpA in control HeLa cells or CpA-TAP (S-tag ChIP), followed by CENP-C probe on Western. Co-migration of beads (Protein G Sepharose, GE Healthcare Cat #17-0618-02) and Ab = S-tag antibody near CpA-TAP protein. b) Whole cell extracts from untransfected control HeLa cells, cells expressing GFP-CpA and CpA-TAP were probed with CENP-A (red) and anti-S-tag (green, CpA-TAP). c) Ratio of CpA-TAP from total extracted chromatin (input) normalized against native CpA. Bars = SEM. d) CpA-TAP on a TAU gel/Western stained with Coomassie and duplicate TAU-Western probing against native CpA and CpA-TAP (using anti-S-tag antibody).


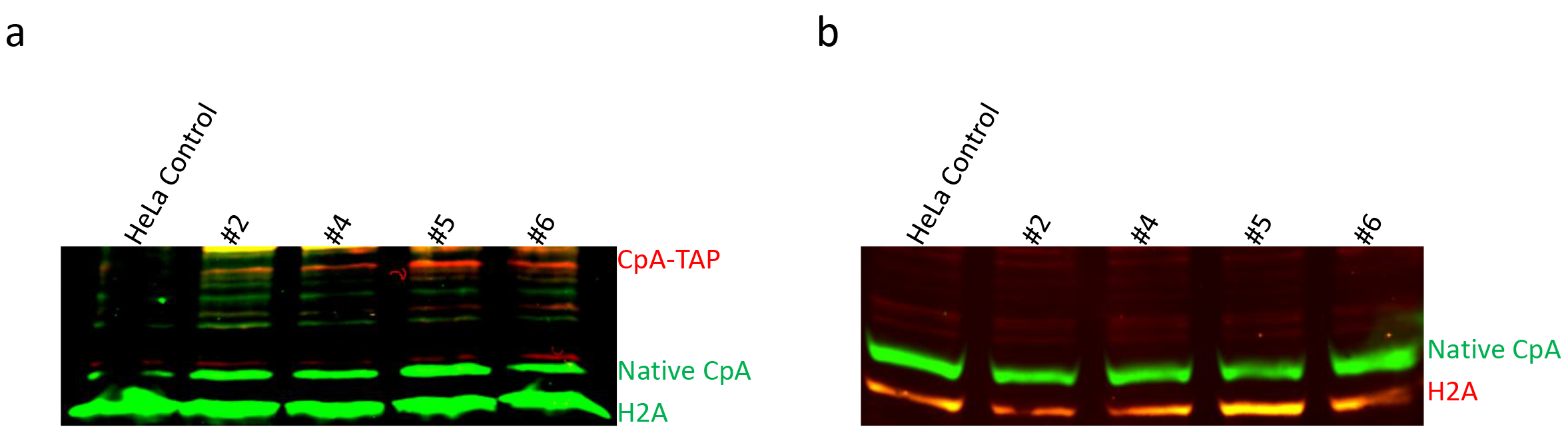


Fig. S2: Knock-in of TAP-tag downstream of the native CpA gene yielded viable heterozygotes only in the DAXX knock-out background. a) Whole cell extracts (WCE) of HeLa control and four viable CpA-TAP knock-in colonies co-expressing both CpA-TAP and native CpA, done in April 2023. b) Same experiment done in A but in September 2023 with no detectable CpA-TAP.

Fig. S3: ChIP’ing native CpA versus tagged CpAs for H1.5 interactions in mono-nucleosomes. a) ChIP native CpA for H1.5 interaction. b) ChIP native CpA versus GFP-CpA. c) ChIP native CpA versus GFP-CpA versus GFP-H3 (twice the amount of anti-GFP antibody). HC = heavy chain.

Fig. S4: Newly loaded SNAP-CpA has
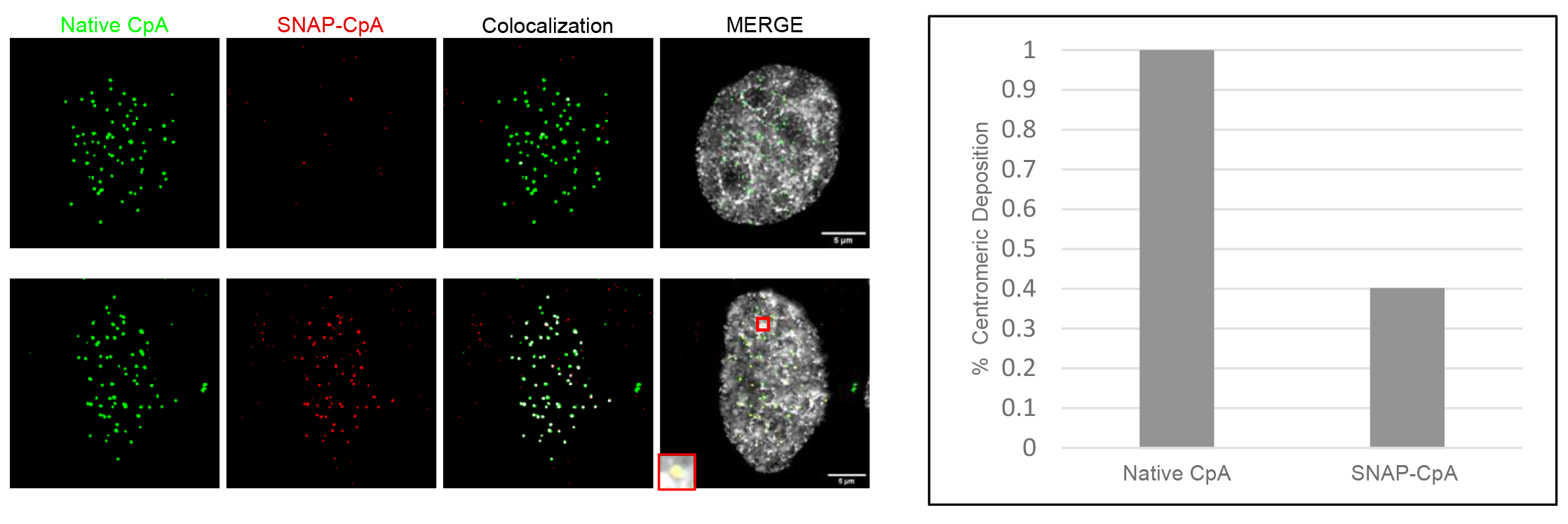
 reduced early G1 phase deposition. Cells were transfected with SNAP-CpA and assessed for coIF with native CpA. Total number of cells with native CpA (green) versus total percentage of those colocalized SNAP-CpA (red) cells that coIF with native CpA (total n = 200) post- early G1 phase.
